# The Impact of HLA-A29 Homozygosity and of the Second HLA-A Allele on Susceptibility and Severity of Birdshot Chorioretinitis

**DOI:** 10.1167/iovs.65.13.47

**Published:** 2024-11-21

**Authors:** Jordan Loeliger, Romain Lhotte, Sahar Gelfman, Eli A. Stahl, Dominique Monnet, Valentin Clichet, Linda Imikirene, Souhila Kecili, Jean-Luc Taupin, Thierry Tabary, Jacques H. M. Cohen, Antoine P. Brézin

**Affiliations:** 1 University Hospital Basel, Department of Ophthalmology, Basel, Switzerland; 2 Université Paris-Cité, Laboratoire d'Immunologie et Histocompatibilité, INSERM U976 IRSL, Hôpital Saint-Louis, Paris, France; 3 Regeneron Genetics Center, Tarrytown, New York, United States; 4 Université Paris Cité, Centre d'ophtalmologie de l'Assistance Publique, Hôpitaux de Paris, Paris, France; 5 University of Reims Champagne-Ardenne, Reims, France

**Keywords:** birdshot chorioretinitis, HLA-A29, HLA-Aw19, HLA-A32

## Abstract

**Purpose:**

HLA-A29 is the main susceptibility factor for birdshot chorioretinitis (BSCR). Our study assessed the impact of the second HLA-A allele alongside HLA-A29 on BSCR severity and susceptibility, focusing on HLA-A29 homozygous patients and those with alleles from the HLA-Aw19 group.

**Methods:**

We included 120 additional cases to our previous analysis of 286 patients with BSCR, all HLA-A29 positive. Patients were categorized based on the second allele being also HLA-A29 (A29/nonA29 vs. A29/A29) or belonging to the HLA-Aw19 family, including A29, A30, A31, and A33 (A29/nonAw19 vs. A29/Aw19). HLA-A32 was analyzed separately (A29/nonA32 vs. A29/A32). The prevalence of these groups among patients with BSCR was compared with their frequencies in a sample of 151,997 French subjects. Disease severity was approximated by assessing disease onset and visual function at the last visit and was compared between patient groups.

**Results:**

When comparing the HLA-A29-positive patients with BSCR to HLA-A29-positive French subjects, we found an overrepresentation of HLA-A29 for the second HLA-A allele (χ² = 4.34; *P* = 0.037; odds ratio, 1.57; confidence interval, 1.01–2.44). Within the HLA-Aw19 broad antigen family, HLA-A32 was found to be under-represented (χ² = 6.15; *P* = 0.013; odds ratio, 0.40; confidence interval, 0.19–0.85). The nature of the second HLA-A allele did not impact disease severity.

**Conclusions:**

Homozygosity for HLA-A29 increased the risk of developing BSCR without affecting disease severity. The under-representation of HLA-A32 in patients with BSCR suggests a potential protective role.

Birdshot chorioretinitis (BSCR) is a rare posterior uveitis characterized by inflammation of the choroid and retina. Although the exact cause of BSCR remains elusive, its link with the HLA-A29 allele has been demonstrated more than 40 years ago.^1^ This association represents the strongest link between an HLA class I antigen and any disease making it unique from an immunogenetic perspective.^2^ Some clinicians are of the opinion that all individuals affected by BSCR are HLA-A29 positive and have advocated the fact that HLA-A29 positivity is a required criterion for the diagnosis of BSCR.^3^ In addition, certain alleles of the endoplasmic reticulum aminopeptidases ERAP1 and ERAP2, involved in the peptide processing machinery of the major histocompatibility complex (MHC) I pathway, are more prevalent in patients with BSCR compared with healthy controls.^4^^,^^5^ We have recently shown that the second HLA-A allele also modulates disease susceptibility to BSCR.^6^ Specifically, carrying a second allele from the Aw19 broad serological family (HLA-A29, -A30, -A31, or -A33), increased disease susceptibility, although one Aw19 member, HLA-A32, was significantly depleted in patients with BSCR compared with A29-positive controls.^6^

The combination of HLA-A29 and HLA-A29 or HLA-Aw19 on the second allele, as well as certain ERAP1/2 alleles, modulate to a higher risk of developing the disease than each gene alone, underscoring their collaborative role. Therefore, a pattern of increased processing combined with increased presentation of ERAP2-specific peptides emerged and suggested a mechanism in which exceeding a peptide presentation threshold activates the immune response in choroids of A29 carriers. It remains unclear whether having two HLA-A29 alleles, thus being homozygous, leads to a more severe manifestation.

In this study, we added 120 patients with BSCR to our previously sequenced cohort. Our primary goal was to analyze if the alleles implicated in the susceptibility for BSCR also played a role in the severity of the disease. In particular, we assessed whether the HLA-A29 homozygous patients had more severe disease manifestations than those which were heterozygous. Moreover, we used the sequencing data from a large sample of the French population to validate and extend our previous findings regarding the role of the second HLA allele in disease susceptibility.

## Methods

### Study Subjects, Main Outcome Measures, and DNA Samples

Our study was based on a dataset of 286 patients with BSCR, for whom sequencing data had been reported previously.^6^ To expand this dataset, the Regeneron genetics center conducted the sequencing of DNA samples from an additional set of 120 BSCR cases to a total of 406 patients. All patients were recruited from the CO-BIRD cohort (ClinicalTrials.gov Identifier: NCT05153057) at a single institution, the Hôpital Cochin in Paris, France. Our clinical data collection methods have been documented already.^7^^–^^10^ The disease diagnosis was based on the criteria defined by an International Consensus Conference and further confirmed by the Standardization of Uveitis Nomenclature group.^11^ In addition, HLA-A29 positivity was a supplementary inclusion requirement for all study patients.

The main outcome measures were the effect of HLA-A29 homozygosity vs. heterozygosity on the susceptibility and severity of BSCR. In this context, all combinations of A29 suballeles were categorized as homozygous.

To investigate whether HLA-A29 homozygous individuals have a higher likelihood of developing BSCR than HLA-A29 heterozygous individuals, we assessed the frequency of homozygous individuals in our BSCR cohort and compared it to the expected frequency of HLA-A29 homozygotes among HLA-A29–positive individuals. To estimate the frequency of the HLA-A29 allele and HLA-A29 homozygous, as well as heterozygous individuals in the French population, we used next-generation sequencing typing data from different laboratories belonging to the Société Francophone d'Histocompatibilité et d'Immunogénétique (SFHI) Consortium, collected into a single previously published database.^12^ Initially, the database gathered 61,393 typings from 17 French laboratories up to May 2020, and was recently expanded to 151,997 individuals with sequenced HLA-A locus by adding 9 laboratories and collecting data up to December 23, 2022.^12^ Anonymized data originated from the HLA laboratories of 25 French cities.

Additionally, our aim was to investigate whether individuals with a second allele belonging to the Aw19 allele group (A29, A30, A31, A32, and A33) were over-represented in our cohort compared with the SFHI cohort and to assess whether they exhibited a more severe form of the disease. Because A32 was found to be under-represented in patients with BSCR,^6^ we excluded A32 from the Aw19 broad antigen group and tested them separately.

Disease severity was primarily assessed based on two clinical indicators, that is, best-corrected visual acuity (BCVA) and visual field results (mean deviation [MD], foveal threshold, and pattern standard deviation [PSD]) at the last patient visit. Moreover, the age at the time of the first symptoms marking the disease onset was also used as an indicator of disease severity. The methods used for the assessment of the visual function were previously reported.^7^^–^^10^^,^^13^ In brief, the BCVA, was measured on a decimal scale and converted to LogMAR and treated as a continuous variable. A BCVA of 0.01 and 0.001 (decimal notation) represented counting fingers and hand motion vision, respectively. Visual field testing used automated perimetry (Humphrey visual field analyzer - Zeiss-Humphrey, San Leandro, CA, USA) with the Fastpac full-threshold 30-2 program. In patients with advanced VF defects, for whom automated perimetry could not be performed, Goldmann perimetry was used and for the study purposes the MD was entered as −30 dB.^13^ The two global indices, MD and PSD, assessed widespread and localized sensitivity loss, respectively. Missing data were labeled as unknown for each variable and presented in the tables. These cases were excluded from specific statistical analyses.

The methods for DNA isolation and HLA genotyping of the second set of patients with BSCR were identical to those used for the sequencing of the first set and previously reported.^6^

### Statistical Analysis

Statistical analyses were performed using R statistical software. A *P* value of 0.05 or less was considered as statistically significant. The statistical analysis on the data matrices involved χ^2^ tests and odds ratio (OR) calculations including lower confidence interval (LCI) and upper confidence interval (UCI). Depending on the distribution and homogeneity of variance, either *t*-tests or Wilcoxon tests were used to compare patient groups. Missing data were labeled as unknown for each variable and displayed in the tables.

## Results

### HLA-A29 and HLA-Aw19 Positivity and Allele Frequency in the French Population

In our centralized next-generation sequencing database of 151,997 individuals from the French population, 15,053 (9.9%) were HLA-A29 positive (Supplementary Table S1). Regarding the suballeles, 14,363 (9.14%) were positive for A29:02 and 1150 (0.75%) were positive for A29:01. Among A29 positive individuals, 14,524 (96.5%) were heterozygous for A29 (N_A29/nonA29_) and 529 (3.5%) were homozygous (N_A29/A29_). The allele frequency (2N_A29/__A29_ + N_A29/nonA29_ / 2N) among the A29 serological group was found to be 5.1%, derived from the contributions of A*29:02 (4.73%) and A*29:01 (0.37%). The other A29 alleles were less frequent, each accounting for less than 0.02%. Among the 15,053 individuals with at least one A29 allele, 1926 (N_A29/Aw19_, 12.8%) carried a second allele belonging to the Aw19 group (A29, A30, A31, and A33); 631 (N_A29/A32_, 4.2%) individuals had a A32 allele as a second HLA-A allele.

### Impact of HLA-A29 and HLA-Aw19 Status on BSCR Susceptibility

This study enrolled 406 HLA-A29 positive patients with BSCR, and their demographic characteristics are summarized in Table 1. Our cohort consisted of 246 females (60.6%) and 160 males (39.4%). The mean age at the time of the first symptoms was 50 ± 10 years and the mean age at diagnosis was 52 ± 10 years.

**Table 1.** Demographic Characteristics and HLA-A29 Status of the 406 Patients With BSCR (*N* = 406)

Characteristic	No. (%) or Mean ± SD
Sex	
Females	246 (60.6)
Males	160 (39.4)
Age at first symptoms	
Mean	50 ± 10
Unknown	5
Age at diagnosis	
Mean	52 ± 10
Unknown	24
Years from symptoms to diagnosis	
Median (IQR)	0.9 (0.2–2.5)
Unknown	28
HLA-A29 status	
HLA-A29 positive	406 (100)
Heterozygotes	
A29:02/nonA29	370 (91.1)
A29:01/nonA29	11 (2.7)
A29:10/nonA29	3 (0.7)
Homozygotes	
A29:02/A29:02	21 (5.2)
A29:02/A29:01	1 (0.3)
Aw19 group status	
A29/A29	22 (5.4)
A29/A30	19 (4.7)
A29/A31	12 (3.0)
A29/A32	7 (1.7)
A29/A33	11 (2.7)
A29/nonAw19	335 (82.5)

Among our patient population, 384 (94.6%) were identified as HLA-A29 heterozygous, and 22 (5.4%) were HLA-A29 homozygous. Within the group of HLA-A29 heterozygous patients, there were 370 patients carrying the A*29:02 allele, 11 patients with the A*29:01 allele, and 3 patients with A*29:10. In the homozygous group, one patient carried both an A*29:02 and an A*29:01 allele, while the remaining patients were homozygous for A*29:02. Furthermore, 65 patients (16%) had an Aw19 group allele (A29, A30, A31, or A33) as their second HLA-A allele, whereas 7 patients (1.7%) had A32 as their second HLA-A allele.

We conducted a comparison between the observed genotype frequency in our BSCR cohort and HLA-A29 positive controls from the SFHI cohort (Table 2). The HLA-Aw19 alleles as a group (A29, A30, A31, and A33) showed a χ^2^ value of 3.76 with a *P* value of 0.052, but were not close to significance when A29 was excluded or considered individually. For HLA-A29, we found a χ^2^ value of 4.34 with a *P* value of 0.037, indicating an increased risk of BSCR in homozygous individuals (OR, 1.57; CI, 1.01–2.44). Additionally, HLA-A32 showed a χ^2^ value of 6.15 with a *P* value of 0.013, indicating an under-representation of this allele in patients with BSCR (OR, 0.40; CI, 0.19–0.85). Although nominally significant, these results did not pass the threshold of multiple test correction (Bonferroni method, *P*_adjusted_ = 8.3e-03) and will require further validation with additional case cohorts.

**Table 2.** Frequency of the Second Allele in Patients With BSCR and in HLA-A29–Positive Individuals From the SFHI Cohort

Genotype	Patients With BSCR (*N* = 406)	SFHI Cohort (*N* = 15,053)	χ^2^	*P* Value	OR [LCI–UCI]
A29/A29	22 (5.4)	529 (3.5)	4.34	0.037	1.57 [1.01–2.44]
A29/A30	19 (4.7)	702 (4.7)	0.00	0.987	1.00 [0.63–1.60]
A29/A31	12 (3.0)	404 (2.7)	0.11	0.734	1.10 [0.62–1.98]
A29/A32	7 (1.7)	631 (4.2)	6.15	0.013	0.40 [0.19–0.85]
A29/A33	11 (2.7)	291 (1.9)	1.29	0.256	1.41 [0.77–2.60]
A29/Aw19	65 (16)	1’926 (12.8)	3.76	0.052	1.30 [0.99–1.70]

LCI, lower confidence interval; UCI, upper confidence interval.

Values are number (%) unless otherwise noted.

Aw19 = A29 or A30 or A31 or A33, with the exclusion of A32.

### Impact of HLA-A29 and HLA-Aw19 Status on BSCR Severity

**Figure 1.**
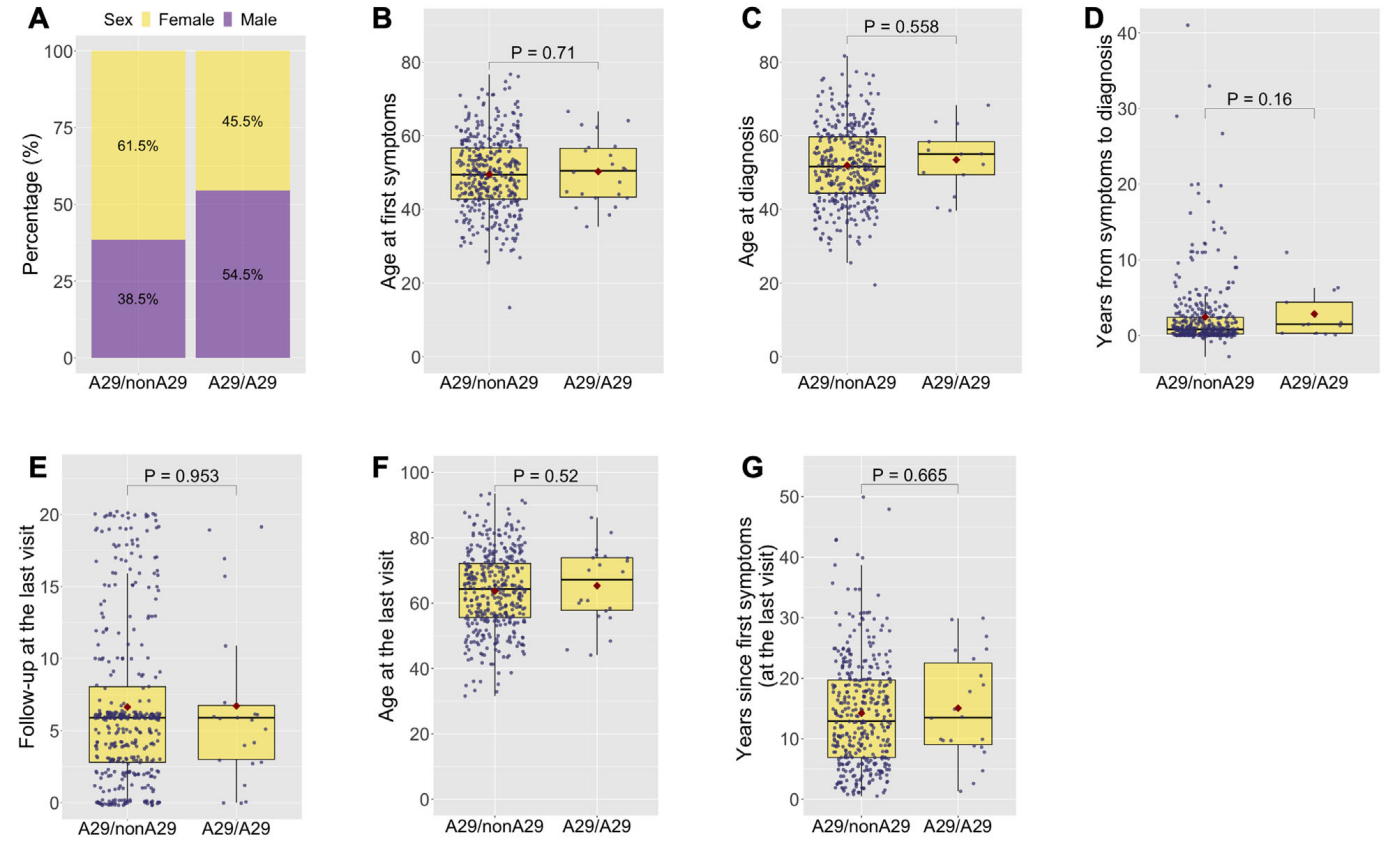
Boxplots corresponding with Table 3, illustrating the distribution of demographic characteristics among HLA-A29 heterozygous (A29/nonA29) and homozygous (A29/A29) patients. Each data point represents an individual patient, with the median depicted by the line inside the box, and the mean highlighted by a red point, and the exact *P* values are displayed on top.

Table 3 and Figure 1 summarize the demographic characteristics of all patients grouped according to their HLA-A29 status. Among HLA-A29 heterozygous patients, 61.5% (236/384) were females, while in HLA-A29 homozygous patients, the proportion of females was 45.5% (10/22). The mean age at the first visual symptoms was 50 ± 9 for HLA-A29 homozygous patients and 50 ± 10 for heterozygous patients, and the mean ages at diagnosis was 52 ± 10 and 53 ± 9, respectively. The time elapsed from the first symptoms to the diagnosis showed a median of 0.8 years (0.2–2.4 years) for heterozygous and 1.5 years (0.3–4.4 years) for homozygous patients. At the last visit, the follow-up was 6.6 ± 5.7 years for heterozygous and 6.7 ± 5.9 years for homozygous patients. The mean age was 64 ± 12 years for HLA-A29 heterozygous and 65 ± 11 years for homozygous patients. The mean time elapsed since the onset of first visual symptoms was 14 ± 9 years for heterozygous patients and 15 ± 9 years for homozygous patients. Overall, no statistically significant differences were observed in any of these parameters.

**Table 3.** Demographic Characteristics of HLA-A29 Heterozygous (A29/nonA29) vs. Homozygous (A29/A29) Patients

	HLA-A29		
Characteristic	A29/NonA29 (*N* = 384)	A29/A29 (*N* = 22)	*P* Value
Sex			0.1^*^
Females	236 (61.5)	10 (45.5)	
Males	148 (38.5)	12 (54.5)	
Age at first symptoms			0.7^†^
Mean ± SD	50 ± 10	50 ± 9	
Unknown	5	0	
Age at diagnosis			0.6^†^
Mean ± SD	52 ± 10	53 ± 9	
Unknown	15	9	
Years from symptoms to diagnosis			0.2^‡^
Median (IQR)	0.8 (0.2–2.4)	1.5 (0.3–4.4)	
Unknown	19	9	
Follow-up^§^			>0.9^†^
Mean ± SD	6.6 ± 5.7	6.7 ± 5.9	
Unknown	1	0	
Years since first symptoms^§^			0.7^†^
Mean ± SD	14 ± 9	15 ± 9	
Age at the last visit^§^			0.5^†^
Mean ± SD	64 ± 12	65 ± 11	

Values are number (%) unless otherwise noted.

* Pearson's χ2 test.

† Welch two Sample t-test.

‡ Wilcoxon test.

§ At the last visit of each patient.

**Figure 2.**
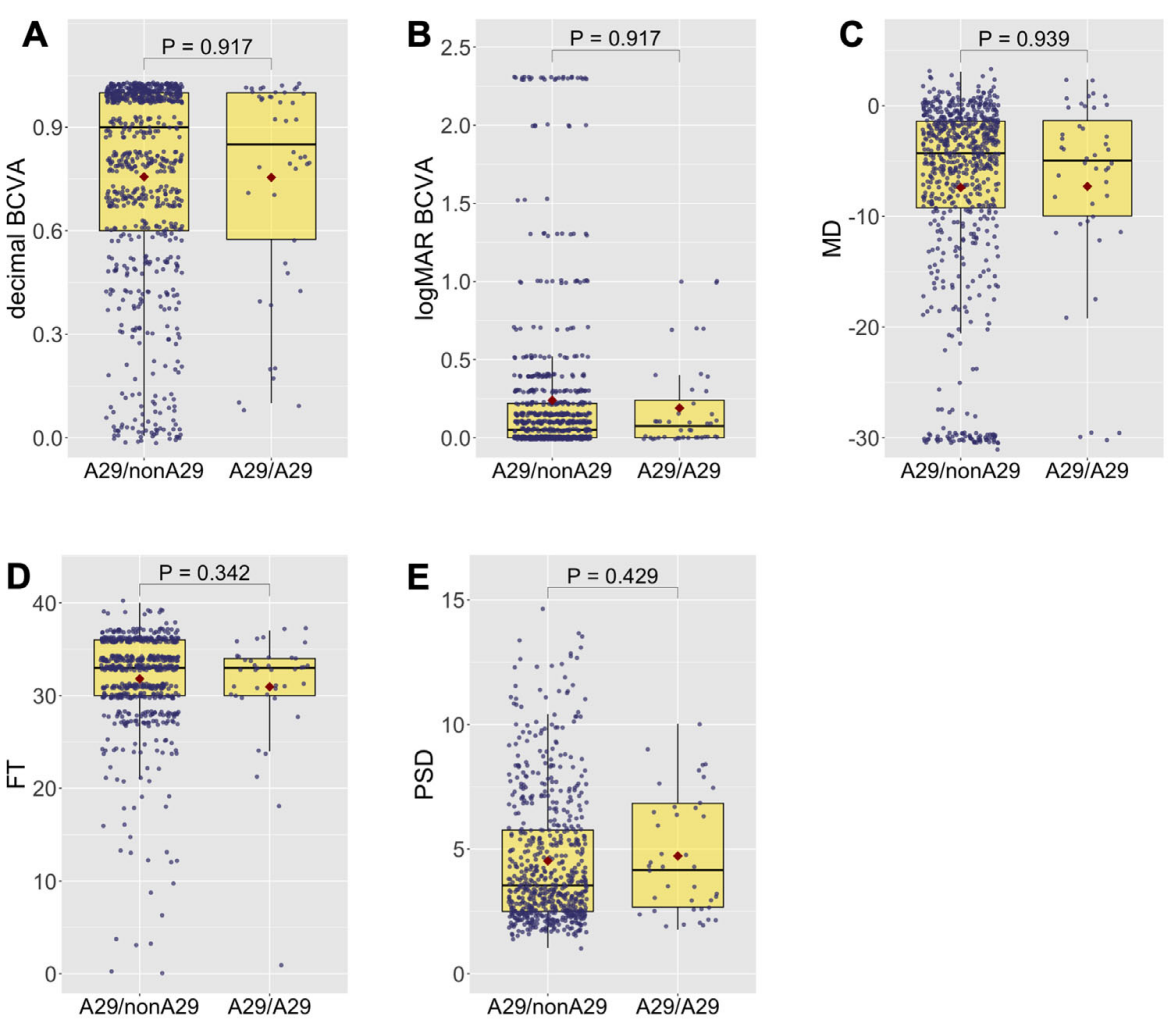
Boxplots corresponding with Table 4 illustrate the visual outcomes among HLA-A29 heterozygous (A29/nonA29) and homozygous (A29/A29) patients at the last visit. Each data point represents a patient's eye, with the median depicted by the line inside the box, and the mean highlighted by a red point, and the exact *P* values are displayed on top.

The severity of the disease was determined by evaluating its impact on visual function at the last patient visit. To this end, the results of visual acuity and visual field testing in HLA-A29 heterozygous and homozygous patients were assessed (Table 4 and in Fig. 2). Between these groups of patients, there was no statistical difference among the parameters which included the BCVA, the MD, the foveal threshold, or the PSD. This was also the case for the comparisons between A29/nonAw19 and A29/Aw19 patients and for A29/nonA32 and A29/A32 patients (data not shown).

**Table 4.** Visual Function of HLA-A29 Heterozygous (A29/nonA29) vs. Homozygous (A29/A29) Patients

	HLA-A29		
	A29/NonA29 (*N* = 768 Eyes)	A29/A29 (*N* = 44 Eyes)	*P* Value^2^
Decimal BCVA			>0.9
Mean ± SD	0.76 ± 0.30	0.75 ± 0.30	
Median (IQR)	0.90 (0.60–1.00)	0.85 (0.58–1.00)	
Unknown	7	0	
LogMAR BCVA			>0.9
Mean ± SD	0.24 ± 0.49	0.19 ± 0.29	
Median (IQR)	0.05 (0.00–0.22)	0.08 (0.00–0.24)	
Unknown	7	0	
MD			>0.9
Mean ± SD	−5.3 ± 5.7	−5.0 ± 5.2	
Median (IQR)	−3.6 (−7.4 to −1.3)	−4.5 (−8.3 to −0.1)	
Unknown	81	4	
FT			0.3
Mean ± SD	31.8 ± 5.3	31.0 ± 6.3	
Median (IQR)	33.0 (30.0–36.0)	33.0 (30.0–34.0)	
Unknown	123	4	
PSD			0.4
Mean ± SD	4.53 ± 2.66	4.73 ± 2.38	
Median (IQR)	3.55 (2.50–5.76)	4.16 (2.68–6.84)	
Unknown	123	4	

BCVA, best corrected visual acuity; FT, foveal threshold; MD, mean deviation; PSD, pattern standard deviation.

* Wilcoxon rank sum test.

## Discussion

In our previous genome-wide association study, we found that, along with HLA-A29, certain second HLA-A alleles were associated with a greater risk of developing BSCR, and those with the largest effects belonged to the same HLA-Aw19 broad antigen serological group: HLA-A29, -A30, -A31, and -A33.^6^ Notably, HLA-A32 exhibited depletion in patients with BSCR compared with A29-positive controls. Among the 286 patients with BSCR sequenced initially, 3.5% exhibited a second HLA-A*29:02 allele, whereas among 108 HLA-A29–positive healthy controls, 0.93% were HLA-A*29:02 homozygotes. Compared with these HLA-A29–positive healthy controls, HLA-A29 alone was not enriched significantly as a second allele of patients with BSCR (OR, 4.44; *P* = 2.2e-03), but as a group, HLA-Aw19 alleles were. Compared with HLA-A29 positive controls from three other large European ancestry control populations, an enrichment of all HLA-Aw19 alleles except A32 was found in BSCR cases.

In this study, we expanded our initial cohort by an additional 120 patients with BSCR and primarily aimed to investigate the impact of the HLA-A genotype on disease severity. However, with an increased sample size, we also revisited our initial analysis and compared the genotype frequencies with a large cohort from the French population to validate and extend our previous findings. To this end, we based our analyses on HLA sequencing data from the largest dataset of French HLA-typed individuals, which provided valuable HLA-A allele and genotype frequencies. Among French individuals, 9.9% were HLA-A29 positive, which reflected a higher prevalence than the previous 7.0% estimate.^2^ Among 15,053 HLA-A29 positive individuals, 3.5% had HLA-A29, 12.8% an HLA-Aw19, and 4.2% HLA-A32 as their second HLA-A allele. Within our cohort of 406 HLA-A29–positive patients with BSCR, 5.4% had HLA-A29, 16% had an HLA-Aw19 group allele, and 1.7% HLA-A32 as the second HLA-A allele. An enrichment of HLA-A29 as the second allele (homozygous patients) was observed in patients with BSCR indicating that having two HLA-A29 alleles corresponds with an increased risk of developing BSCR. Furthermore, HLA-A32 depletion in patients with BSCR compared with A29-positive healthy individuals was found. In the current study, HLA-Aw19 alleles as a group were close to reaching statistical significance as the second allele in patients with BSCR. However, this effect was primarily driven by HLA-A29, because other individual HLA-Aw19 alleles were not significantly enriched as the second allele. Compared with our previous study, which involved comparisons with multiple cohorts, the overall enrichment of the HLA-A29 allele as the second allele was more pronounced, while the enrichment of HLA-Aw19 alleles appeared weaker. This difference is likely due to the larger cohort of sequenced patients with BSCR and the use of a different comparison cohort, as detailed elsewhere in this article. Consistent with our previous findings, HLA-A32 was significantly depleted in patients with BSCR compared with A29-positive healthy individuals.

The association of BSCR with endoplasmic reticulum aminopeptidase 2 (ERAP2) suggests a pivotal role of antigen processing and presentation in BSCR's pathogenesis.^14^ Therefore, we aimed to interpret our results within the context of how ERAP2-processed antigens are presented to the immune system.

From a quantitative perspective, HLA-A29 homozygotes likely express higher levels of HLA-A29 molecules per cell compared with HLA-A29 heterozygotes. This finding might be of relevance because HLA-A29 alleles are low expressed alleles compared with other HLA-A alleles.^15^ Thus, an increased presentation of ERAP2-derived peptides on a larger amount of HLA-A29 molecules may more readily exceed a peptide presentation threshold, triggering a T-cell response and BSCR development.

From a qualitative perspective, HLA molecules derived from the Aw19 group alleles are similar structurally and belong to the same supertype.^16^ Given that structural similarity highly correlates with peptide binding specificity, HLA molecules derived from Aw19 allele group may present similar peptides. However, compared with HLA-A29, all Aw19 group alleles show amino acid mismatches in the primary anchor residues of the peptide binding pocket, which could affect peptide binding specificity.

The significant depletion of HLA-A32 in patients with BSCR indicates a potential protective mechanism among HLA-A29–positive individuals, although the exact explanation remains unclear. Prediction of HLA-A32– and HLA-A29–associated peptides based on a large peptidome dataset suggests that HLA-A32:01 exhibits a certain degree of flexibility at its C-terminal anchor, allowing it to accommodate a variety of residues, including a preference for tyrosine (Y) at position 9.^17^ This finding suggests that HLA-A32:01 may be capable of binding similar peptides as HLA-A29:02. Therefore, one hypothesis is that HLA-A32 may competitively bind pathogenic peptides, decreasing their availability for presentation by HLA-A29 and thereby preventing the activation of autoreactive T cells. The same peptides could adopt different conformations when bound to HLA-A32 compared with HLA-A29, altering their interaction with T-cell receptors. This difference in peptide–MHC conformation might result in a T-cell recognizing the peptide on HLA-A29, but not more or less effectively, on HLA-A32, leading to either no recognition at all, partial activation, or anergy, thereby preventing a full-blown auto-aggressive immune response. This decoy function could help to prevent pathogenic immune responses.

Alternatively, during the development of the immune system, tolerance induction occurs as proteins specific to the eye are presented in the thymus.^18^ The affinity with which thymocyte T cell receptors bind self-peptide–MHC complexes determines their fate during thymic selection. High-affinity binding typically results in negative selection and apoptosis, eliminating self-reactive T cells. HLA-A32 might provide more proficient antigen presentation, favoring the negative selection and apoptosis of specific self-reactive T cells, thus contributing to central tolerance. A similar mechanism has been postulated for the protective effect of HLA-DRB1*13 in autoimmune diseases.^19^

HLA-A32 could also promote the development of regulatory T cells (Tregs), which play a critical role in maintaining immune tolerance and suppressing autoreactive T cells.^20^ During thymic selection, some thymocytes that recognize self-peptides with intermediate affinity are not eliminated but instead differentiate into Tregs.^20^ These Tregs then leave the thymus and help to maintain immune tolerance in the periphery by suppressing immune responses that could target self-tissues. Additionally, HLA-A32 may contribute to peripheral tolerance by presenting peptides in a manner that promotes immune regulation, such as inducing T-cell anergy or expanding Tregs in peripheral tissues. However, because eye-specific antigens are sequestered behind the blood–retinal barrier, contributing to the immune privilege, the development of peripheral tolerance might not be as efficient.^21^

Understanding these potential protective pathways underscores the need for further research to fully elucidate their therapeutic potential. If the inciting antigens were identified, future studies could also explore the efficacy of inducing tolerance in patients with BSCR by administering these specific antigens.^20^ However, the main challenge is the requirement for precise knowledge of the causative peptides.

We observed a greater number of males than females among homozygous patients. This finding is contrary to the typical pattern where females are affected more commonly than males.^13^ However, this difference was not statistically significant. We went on to assess the severity of the disease by analyzing the results of visual acuity and visual field testing at the last visit. Importantly, the mean disease duration, reflected by the time elapsed since the first symptoms, and the age at the visit, were comparable among HLA-A29 heterozygous and homozygous patients at the last visit, ensuring good comparability of the severity. Visual acuity and visual field results were very similar between HLA-A29 homozygous and heterozygous patients, despite consistently, albeit minimal worse results in homozygous patients. Moreover, we included the age at onset of the first symptoms as an additional marker of disease severity. Because visual impairment progressively worsens over time in patients with BSCR,^13^ an earlier onset is likely associated with a poorer prognosis. However, the disease onset seemed to be similar for HLA-A29 homozygous and heterozygous patients; both groups had a mean onset of first symptoms at 50 years old and a mean age at diagnosis of 52 years old.

The comparable outcomes in visual acuity and visual field testing, along with the similar age at disease onset, suggest a similar severity of BSCR between these two groups. Similarly, individuals with or without HLA-A32 as their second allele demonstrated comparable disease severity.

Several limitations should be considered when interpreting the findings of this study. First, we compared groups (patients with BSCR vs. French individuals) with substantially different sample sizes, which may affect the power of our comparisons negatively. This factor is particularly relevant given that BSCR is a rare disease and, despite analyzing what we believe to be the largest cohort to date, the overall sample size remains relatively small. This factor additionally limits statistical power and makes it harder to detect true associations. As a result, achieving significance, particularly after correcting for multiple comparisons, becomes more challenging. Although our results are nominally significant, they do not pass the threshold for multiple test correction. Consequently, future studies with even larger sample sizes and additional case cohorts will be necessary to validate these findings. However, our approach highlights potentially clinically relevant associations for further investigation, which would be masked if we relied solely on *P* values meeting strict, corrected significance levels, especially given the relatively small number of comparisons made. Second, we did not investigate the impact of different treatment methods, because there is substantial heterogeneity in treatment history. Analyzing treatment effects at the last visit is challenging due to the variability in previous treatments and the fact that some patients were followed at other centers before coming to ours. Although we aim to treat all patients with BSCR according to similar principles, treatment decisions are influenced by numerous factors, including whether a patient requires treatment, experiences side effects that lead to discontinuation, or is reluctant to accept the prescription of immunosuppressive therapy, making it difficult to draw any conclusions.

To our knowledge, this study is the first to examine the frequency of HLA-A29 homozygosity and its impact on disease severity in a large cohort of patients with BSCR. However, similar investigations have been conducted regarding HLA-B27 and its influence on the susceptibility and severity of ankylosing spondylitis. In one Finnish study, the prevalence of HLA-B27 homozygosity among patients with ankylosing spondylitis was significantly higher than expected, based on the Hardy–Weinberg equilibrium.^22^ Additionally, the study showed a significant association between HLA-B27 homozygosity and an earlier age of symptom onset and diagnosis in ankylosing spondylitis patients. More broadly, homozygosity for certain HLA alleles has been associated with increased susceptibility in autoimmune diseases such as systemic lupus erythematosus,^23^ common variable immunodeficiency,^24^ and challenges in clearing hepatitis B virus infections.^25^

## Conclusions

HLA-A29 homozygosity is associated with an increased risk of developing BSCR compared with HLA-A29 heterozygosity, but otherwise does not significantly affect clinical manifestations. The findings of this study support a model wherein increased presentation of ERAP2-specific peptides on HLA-A29 a peptide presentation threshold leading to the activation of an immune response and development of BSCR. Importantly, our data suggest that, once the immune response is initiated, the presence of a second HLA-A29 allele does not have a discernible influence on the disease course or severity. Furthermore, the significant depletion of HLA-A32 in our cohort suggests that it might have a protective role.

## Supplementary Material

Supplement 1
