# Supplementary material for: The Impact of HLA-A29 Homozygosity and of the Second HLA-A Allele on Susceptibility and Severity of Birdshot Chorioretinitis
Source: Invest Ophthalmol Vis Sci. 2024 Nov 21;65(13):47. doi: 10.1167/iovs.65.13.47 (PMC11585056; doi:10.1167/iovs.65.13.47)
Supplement: Supplement 1 [file iovs-65-13-47_s001.pdf]

SUPPLEMENTARY MATERIAL

The impact of HLA-A29 homozygosity and of the second HLA-A allele on susceptibility and severity of birdshot chorioretinitis

Jordan Loeliger *et al.*

Supplementary Table 1: HLA typing data from NGS sequencing of 151'997 individuals: **A**) Percentage of individuals positive for a specific HLA-A allele group arranged by decreasing frequency. **B**) Detailed analysis of HLA-A29 suballeles, including counts, allele frequency, percentage of a specific suballele among all HLA-A29 suballeles, and positivity among individuals.

A

Assigned HLA-A allel group	Percentage of positive individuals (%)
A2	46.7
A1	22.8
A3	22.7
A24	18.3
A11	11.3
A29	9.9
A68	8.9
A30	8.8
A32	7.4
A26	6.9
A23	6.6
A31	5.0
A33	4.8
A25	2.9
A66	1.4
A34	1.0
A74	1.0
A36	0.4
A80	0.3
A69	0.3
A43	0.0
Total	187.2

B

HLA-A29	Count	Locus (%)	First-field (%)	Individuals (%)
A29:02	14'363	4.725	92.177	9.14
A29:01	1'150	0.378	7.38	0.748
A29:10	27	0.009	0.173	0.018
A29:95	18	0.006	0.116	0.011
A29:09	6	0.002	0.039	0.004
A29:44	3	< 0.001	0.019	0.002
A29:11	2	< 0.001	0.013	0.001
A29:50	2	< 0.001	0.013	0.001
A29:26	2	< 0.001	0.013	< 0.001
A29:04	1	< 0.001	0.006	< 0.001
A29:104	1	< 0.001	0.006	< 0.001
A29:12	1	< 0.001	0.006	< 0.001
A29:134	1	< 0.001	0.006	< 0.001
A29:15	1	< 0.001	0.006	< 0.001
A29:40	1	< 0.001	0.006	< 0.001
A29:45	1	< 0.001	0.006	< 0.001
A29:46	1	< 0.001	0.006	< 0.001
Total	15'582	5.12	100	9.925

- Count corresponds to the number of times a distinct allele was found (if an individual is homozygous, it counts twice).
- Locus is Count/(151'997*2) (the percentage of occurrence of this allele within the locus).
- First-field is the same percentage but within the corresponding first field.
- Individuals is the percentage of individuals having this allele at least once.
